# The Role and Mechanism of Hair Follicle Neural Crest Stem Cells and Their Small Extracellular Vesicles in Repair of Peripheral Nerve Injury

**DOI:** 10.1155/sci/1665875

**Published:** 2026-09-29

**Authors:** Jiachen Zheng, Siying Fei, Yang Xu, Chi Zhang, Haiyan Lin, Fang Liu

**Affiliations:** ^1^ Basic Medical College, Naval Medical University, Shanghai, China, smmu.edu.cn; ^2^ Department of Burn Surgery, The First Affiliated Hospital of Naval Medical University, Shanghai, China, smmu.edu.cn; ^3^ Department of Human Anatomy, Naval Medical University, Shanghai, China, smmu.edu.cn

## Abstract

Peripheral nerve injury (PNI) remains a vital clinical challenge due to the limited efficacy of current therapeutic strategies. Autologous nerve grafting, the gold standard for repairing PNI, remains limited by certain donor site morbidity, and alternative approaches include cell therapy, nerve conduits, gene therapy, and electrical stimulation with immune rejection and ethical controversies. Yet, hair follicle neural crest stem cells (hfNCSCs), a pluripotent cell population originating from the embryonic neural crest, have emerged as a promising candidate. hfNCSCs exhibit high differentiation potential, ethical acceptability, and accessibility, possessing significant advantages in axonal regeneration and functional recovery. Concurrently, small extracellular vesicles (sEVs) derived from stem cells have garnered increasing attention for their low immunogenicity, non‐tumorigenicity, and convenient storage. Preclinical studies highlight their synergistic potential in modulating neuroinflammation, enhancing Schwann cell (SC) activity, and promoting nerve repair. This review summarizes recent advances in hfNCSCs and sEVs for PNI repair, with a focus on molecular mechanisms and translational applications. We further discuss the challenges and future directions of hfNCSCs‐sEVs as a cell‐free therapeutic strategy in neural regeneration.

## 1. Background

Peripheral nerve injury (PNI) frequently results from mechanical trauma, iatrogenic interventions, or complications of diseases such as tumor resection with partial or complete impairment of motor and sensory functions and manifesting as persistent numbness, weakness, or even paralysis and often causing neuropathic pain. The resulting physical disability can significantly affect a patient’s quality of life [1, 2]. Pathologically, PNI triggers retrograde axonal degeneration in proximal nerve segments and Wallerian degeneration in distal stumps. Schwann cells (SCs), the principal glial cells of the peripheral nervous system, undergo dedifferentiation after demyelination, forming Büngner bands to guide axonal regrowth while secreting neurotrophic factors (NTFs). However, aging SCs can exhibit an imbalance in stress granule homeostasis, which impairs their axonal regeneration‐supporting function; although partial reprogramming can reverse this phenomenon, the persistence of this defect under natural aging conditions limits regenerative efficiency [3].

Given the limited intrinsic regenerative capacity of injured peripheral nerves, effective clinical interventions are urgently needed. Autologous nerve transplantation, the current clinical “gold standard” for bridging nerve gaps, is limited by finite donor availability, donor denervation, and graft‐host mismatches [4]. Alternatives such as bioengineered nerve conduits and tissue‐engineered constructs have been emerging and have been gradually applied to address these challenges. Within this paradigm, seeding cells, particularly stem cells, have become pivotal due to their capacity to differentiate into neural lineages and to provide a suitable microenvironment and promote axon regeneration by secreting NTFs. Adult stem cells (ASCs) offer advantages in self‐renewal and asymmetric cell division. Yet, their clinical translation is hindered by their isolation, immunosuppression potential, and tumorigenic risks [5]. Recently, hair follicle neural crest stem cells (hfNCSCs), a unique ASC population residing in the hair follicle bulge, have emerged as a promising candidate. Originating from the ectodermal neural crest, hfNCSCs exhibit exceptional pluripotency, differentiating into neurons, glial cells, and Schwann‐like cells [6, 7]. Critically, their autologous sourcing minimizes ethical concerns and immune rejection, promoting axon regeneration and myelination to repair PNI [8].

In parallel with advances in cell‐based therapies, stem cell‑derived small extracellular vesicles (sEVs) have garnered increasing attention as a cell‑free alternative. According to the Minimal Information for Studies of Extracellular Vesicles (MISEV) 2023 guidelines, sEVs are nanosized vesicles enriched with miRNAs, proteins, and lipids [9]. These sEVs mediate intercellular communication by modulating neuroinflammation, promoting SC migration, and stimulating angiogenesis [10]. Notably, stem cell‐derived sEVs (SC‐sEVs) synergistically bypass the risks of direct cell transplantation (e.g., tumorigenicity) while amplifying therapeutic efficacy through targeted molecular delivery [11].

Together, hfNCSCs and their derived sEVs hold important promise for advancing PNI repair. This review synthesizes recent preclinical advances in hfNCSCs and their sEVs for PNI repair, with a focus on molecular mechanisms and translational applications, and discusses the challenges and future directions of hfNCSCs‑sEVs as a cell‑free therapeutic strategy in neural regeneration.

## 2. hfNCSCs

### 2.1. Biological Characteristics of hfNCSCs

hfNCSCs are a pluripotent stem cell population originating from the embryonic neural crest. The discovery of hfNCSCs in adult mammals was first reported in 2004 by Sieber‐Blum et al. [12], who identified neural crest‐derived stem cells within the hair follicle bulge of mice. hfNCSCs are characterized by the expression of neural progenitor markers (Nestin, Sox10) and retain the capacity for differentiation into various cell types, such as neurons, glial cells, osteocytes, melanocytes, smooth muscle cells, and SCs [7, 8].

hfNCSCs offer distinct advantages in accessibility, differentiation efficiency, and cryopreservation stability. Compared to conventional neural stem cells (NSCs), hfNCSCs can be noninvasively harvested from autologous hair follicles, avoiding ethical concerns and enabling large‐scale isolation. hfNCSCs exhibit superior neural differentiation rates relative to ASCs such as bone marrow mesenchymal stem cells (BMSCs). Cryopreserved hair follicles retain hfNCSC viability and pluripotency post‐thawing, facilitating long‐term storage and clinical applications.

These properties, combined with their ability to promote axonal growth and functional recovery in preclinical models, position hfNCSCs as a promising candidate for PNI repair [13, 14]. However, a well‑recognized translational bottleneck should be noted: the absolute cell yield per hair follicle is substantially lower than that from bone marrow or adipose tissue. This low starting cell number requires extensive in vitro expansion to generate sufficient hfNCSCs or their sEVs for therapeutic applications, posing challenges for scalability and cost‑effectiveness.

To provide a comparative overview of key stem cell types used in peripheral nerve regeneration, including their availability, differentiation potential, secretome, and yield, Table 1 summarizes the characteristics of hfNCSCs and other frequently studied stem cells.

**Table 1:** Characteristics of hfNCSCs and other stem cell types.

Stem cell type	Availability [15, 16]	Invasive procedure of collection [15, 16]	Differentiation [7, 8, 17–35]	Secretome [31, 36–50]	Unit basis	Typical primary yield [51–55]
hfNCSCs	High (autologous, minimally invasive biopsy)	Low (local anesthesia, minor procedure)	Neurons, glial cells, osteocytes, melanocytes, smooth muscle cells, and Schwann cells	GDNF, IGF, NGF	Per individual hair follicle (P0)	≈1.65 × 10^5^ cells
BMSCs	Moderate (requires bone marrow aspiration)	High (invasive surgical procedure)	Mesenchymal lineages (fat, bone, muscle, and cartilage), neuronal or glial differentiation, Schwann cells	NGF, BDNF, GDNF, CNTF	Per mL aspirate	2885 ± 1716 CFU‑f
ADSCs	High (abundant autologous tissue, minimally invasive liposuction)	Low (minimally invasive procedure)	Multidirectional differentiation, including toward Schwann‐like cells	BDNF, bFGF, VEGF, PDGF, HGF	Per mL lipoaspirate	≈185 × 10^3^ SVF cells
UCMSCs	Moderate (allogeneic, dependent on donor availability)	Not invasive (collected postdelivery)	Schwann cells, brain cells, and axons	Antiangiogenic factors, CXCL1, CXCL5, CXCL6, CXCL8, HGF, bFGF, VEGF‐D, PDGF‐AA, TGF‐β2, G‐CSF	Per sample (whole cord)	6.5 × 10^6^ ± 0.8 cells/sample
AFMSCs	Moderate (allogeneic, dependent on donor availability)	Not invasive (collected via amniocentesis)	Osteogenic, chondrogenic, adipogenic, myogenic, endothelial, lung, hepatic, or neurogenic lineages, inclusive of all three embryonic germ layers	bFGF, LIF, NGF, BDNF, NT3, GDNF, CDNF, VEGF, HGF	Per liter of fluid	5.8 × 10^6^ ± 0.6 cells/sample
DPSCs	Low (limited autologous source)	Low (extraction during dental procedures)	Neurons, myocytes, hepatocytes, angiogenic and neurogenic potential	NO, TRAIL, CXCL12, BMP4, BMP6	Per tooth	>1 × 10^6^ cells

*Note:* Adapted from Lavorato et al. [15]. This table focuses on the specific characteristics and shows a comparison of the various types. Absolute cell yield per unit tissue varies widely; hfNCSCs typically yield fewer cells per isolation compared to BMSCs or ADSCs.

Abbreviations: ADSCs, adipose‐derived mesenchymal stem cells; AFMSCs, amniotic fluid mesenchymal stem cells; BDNF, brain‐derived neurotrophic factor; bFGF, basic fibroblast growth factor; BMSCs, bone marrow mesenchymal stem cells; BMP, bone morphogenetic protein; CDNF, ciliary‐derived neurotrophic factor; CFU‐f, colony‐forming unit fibroblasts; CNTF, ciliary neurotrophic factor; CXCL, CXC chemokine ligand; DPSCs, dental pulp stem cells; G‐CSF, granulocyte colony‐stimulating factor; GDNF, glial cell line‐derived neurotrophic factor; HGF, hepatocyte growth factor; hfNCSCs, hair follicle neural crest stem cells; IGF, insulin‐like growth factor; LIF, leukemia inhibitory factor; MNC, mononuclear cell; NGF, nerve growth factor; NO, nitrogen oxide; PDGF, platelet‐derived growth factor; PDGF‐AA, platelet‐derived growth factor‐AA; SVF, stromal vascular fraction; TGF‐β2, transforming growth factor‐β2; TRAIL, tumor necrosis factor‐related apoptosis‐inducing ligand; UCMSCs, umbilical cord‐derived mesenchymal stem cells; VEGF, vascular endothelial growth factor; VEGF‐D, vascular endothelial growth factor‐D.

### 2.2. Mechanism of hfNCSCs in PNI Repair

hfNCSCs may promote PNI repair through two synergistic mechanisms (Figure 1): direct differentiation and paracrine modulation.

**Figure 1:**
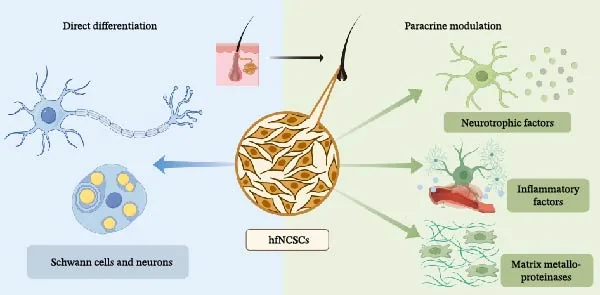
The mechanism of hfNCSCs in PNI repair.

#### 2.2.1. Direct Differentiation Into Functional Cells

hfNCSCs differentiate into SCs and neurons, directly reconstructing damaged neural circuits. Lin et al. [56] bridged 4‐cm‐long sciatic nerve defects in rats using hfNCSCs‐seeded acellular nerve grafts, achieving target differentiation into SCs and functional recovery comparable to autografts. Sakaue and Sieber‐Blum [57] established a detailed experimental protocol demonstrating that human epidermal neural crest stem cells derived from hair follicles can be efficiently and rapidly induced to differentiate into SCs with high purity in vitro. The induced cells consistently expressed specific SC markers and secreted key NTFs associated with nerve repair.

#### 2.2.2. Paracrine Secretion of Regenerative Mediators

hfNCSCs secrete NTFs such as brain‐derived neurotrophic factor (BDNF), glial cell line‐derived neurotrophic factor (GDNF), and neurotrophin‐3 (NT3), which are essential for neuronal survival and functions, stimulating neurogenesis and regrowth of multiple descending axon tracts [46, 47].

Li et al. [58] revealed that at 7 days post‑bridging of a 10 mm sciatic nerve defect in rats, the transplantation of epidermal neural crest stem cells isolated from rat whisker hair follicles significantly reduced the expression of proinflammatory cytokines tumor necrosis factor‑α (TNF‑α) and interleukin‑6 (IL‑6), along with markedly enhanced the expression of anti‑inflammatory cytokines interleukin‑4 (IL‑4) and interleukin‑13 (IL‑13), compared to the DMEM control group (*p* < 0.05). This is consistent with the hypothesis that transplantation of hfNCSCs modulates the local inflammatory milieu by shifting the balance from proinflammatory to anti‑inflammatory mediators, thereby creating a regenerative microenvironment that facilitates nerve repair. Furthermore, metalloproteases (e.g., matrix metallopeptidase‐2, matrix metallopeptidase‐3, and MMP‐14) released by hfNCSCs are likely to participate actively in the remodeling of extracellular matrix during scar formation and thus potentially reduce the barrier effect of the scar and facilitate scar dissolution and axon pathfinding [59].

These dual mechanisms—structural integration and molecular signaling—may enable hfNCSCs to address both morphological and functional deficits in PNI.

## 3. SC‐sEVs

### 3.1. Biological Characteristics of sEVs

sEVs are nanosized vesicles typically characterized by a diameter of less than 200 nm and the presence of specific protein markers, including transmembrane tetraspanins (e.g., CD63 and CD81) and cytosolic proteins involved in vesicle formation (e.g., TSG101) [9]. These features are consistent with an endosomal origin, and sEVs are often released via the multivesicular body pathway. However, definitive evidence of endosomal biogenesis requires experimental demonstration such as immunoaffinity capture of endosomal markers or density gradient purification, which was not performed in the majority of the cited studies. Accordingly, in line with MISEV 2023 guidelines, we use the neutral term “sEVs” throughout this review to maintain terminological precision.

To effectively utilize sEVs for therapeutic purposes, their precise isolation from biological samples is critical. Current primary isolation methods include ultracentrifugation, size‐exclusion chromatography, precipitation kits, immunoaffinity capture, ultrafiltration, microfluidics‐based isolation, and density gradient centrifugation, each with distinct advantages and limitations (Table 2).

**Table 2:** Isolation techniques of sEVs.

Isolation technique	Key principle	Yield	Purity	Scalability	Advantages	Disadvantages	Applicability to hfNCSCs‐sEVs	Ref.
Ultracentrifugation (UC)	Sequential centrifugation steps using high g‐forces (e.g., 100,000 g) to pellet sEVs.	Moderate (sample‐dependent)	Moderate	Moderate	Considered the “gold standard”; has good recovery of particle morphology; no specialized reagents; handles large sample volumes	Time‐consuming (often >5 h); high equipment cost; shear forces can damage sEVs; shows particle aggregation and yields sEVs with smaller mean size	Standard isolation method in current hfNCSCs‐sEVs studies; potential bioactivity loss reflects a general technical limitation of ultracentrifugation.	[60, 61]
Size‐exclusion chromatography (SEC)	Separates particles by size as they pass through a porous column matrix; sEVs elute first.	High	High	Moderate (column capacity limited)	High yield; preserves sEVs structure and function well; less time‐consuming (~0.3 h); low cost	Coisolation of lipoproteins in plasma/serum samples; limited single‐batch processing sample volume	Suitable for functional studies requiring intact bioactivity; caution advised when processing lipoprotein‐rich samples.	[62–64]
Polymer‐based precipitation	Polymers (e.g., PEG) alter solubility, causing sEVs to precipitate out at low speeds.	Moderately high	Moderate	Higher than UC	Simple, fast protocol; no ultracentrifuge required; works with small volumes	High coprecipitation of non‐sEV proteins/contaminants; polymer residue interferes with downstream analysis	Suitable for rapid initial screening, but impurities may interfere.	[65]
Immunoaffinity capture	Antibodies (on beads/columns) bind specific surface markers (e.g., CD63, CD81) on sEVs.	Moderate	High	Low‐moderate	High specificity; isolates subpopulations	High cost; sEV labeling requires screening and optimization; washing may damage the sEV structure	Nanoantibodies derived from naïve libraries enable selective and stable isolation of specific EV subpopulations for research and diagnostics	[66]
Ultrafiltration	Uses membranes with specific pore sizes (e.g., 100–500 kDa) to concentrate and isolate sEVs.	Higher than polymer‐based precipitation	Overall lower purity	High	Fast; simple; low‐cost	Membrane clogging; shear stress may damage sEVs	Not suitable for samples with a high amount of impurities	[67]
Microfluidics‐based Isolation	Separation in microchannels using forces/affinity (acoustic, electrophoretic).	High	High	Very high, highly adaptable for automation	Operating with minute volume of samples and rapid process, in order to isolate sEVs with high yield and high purity	Low throughput; requires specialized equipment	Ideal for precious or limited stem cell samples, or integrated analysis	[67, 68]
Density gradient centrifugation	Separation based on buoyant density in a medium (e.g., iodixanol).	Low‐moderate	High	Low‐moderate	High purity; effectively removes protein contamination	Time‐consuming; expensive equipment; low recovery rate; complex operation	Best for downstream omics or mechanistic studies requiring highest purity	[9]

Among various sources, SC‐sEVs have emerged as a safer and more stable alternative to whole‐cell therapies, widely applied in research (Table 3). Compared to direct stem cell transplantation, SC‐sEVs offer several distinct advantages. First, they lack replicative DNA, eliminating the risk of tumorigenicity, arrhythmias, and microvascular occlusion associated with stem cell engraftment [75]. Second, minimal expression of major histocompatibility complex (MHC) molecules on sEV membranes reduces immune rejection. Third, SC‐sEVs retain bioactivity during long‐term storage at −20°C without potentially toxic cryopreservatives, greatly simplifying manufacturing and distribution [76]. Notably, the vitality and potency of MSC‐sEVs need not be monitored and maintained during storage [77]. Finally, SC‐sEVs are free from the ethical concerns that still surround certain stem cell sources.

**Table 3:** Stem cell‐derived sEVs (SC‐sEVs) for the treatment of PNI models.

sEVs	In vitro model	In vivo model	Findings	Refs.
ADSCs‐sEVs	Cocultures of DRG neurons with ADSCs‐sEVs	Rat sciatic nerve crush injury	Increased mean length of neurites in cocultures; increased axonal regeneration and improved functionality; presence of neural growth factors transcripts	Bucan et al. [69]
ADSCs‐sEVs	DRG neurons cocultured with sEVs‐internalized SCs	Rat sciatic nerve transection model with a 10 mm gap	Promoted SCs proliferation, migration, myelination, and secretion of neurotrophic factors; improved axon regeneration, myelination, and restoration of denervation muscle atrophy	Chen et al. [70]
LPS‐treated BMSCs‐sEVs	An inflammatory model in RAW264.7 cells	Rat sciatic nerve crush injury	Accelerated functional recovery, axon regeneration, remyelination; enhanced M2 macrophage polarization via TSG‐6/NF‐κB/NLRP3 signaling pathway	Li et al. [71]
UCMSCs‐sEVs	N/A	Rat sciatic nerve transection model with a 10 mm gap	Promoted peripheral nerve regeneration and restoration of motor function; observed more CD31‐positive endothelial cells	Tang et al. [72]
DPSCs‐sEVs	A coculture system between DPSCs and SCs	Rat sciatic nerve injury model	Enhanced neurite proliferation, migration, and secretion of neurotrophic factors; improved the walking behavior, axon regeneration, and myelination	Chai et al. [73]
iPSC‐sEVs	N/A	Rat sciatic nerve crush injury model	Enhanced functional recovery, attenuated pain, and accelerated sciatic nerve regeneration	Aldali et al. [74]

*Note:* This table focuses on SC‐sEVs for the treatment of PNI models. The term “sEVs” is used in accordance with MISEV 2023 guidelines; the original studies cited may have used “exosomes.”

Abbreviations: ADSCs‐sEVs, sEVs derived from adipose‐derived mesenchymal stem cells; BMSCs‐sEVs, bone marrow mesenchymal stem cell‐derived sEVs; DPSCs‐sEVs, dental pulp stem cell‐derived sEVs; DRG, dorsal root ganglion; iPSC‐sEVs, sEVs derived from induced pluripotent stem cells; LPS, lipopolysaccharide; UCMSCs‐sEVs, sEVs derived from umbilical cord‐derived mesenchymal stem cells.

### 3.2. Mechanism of SC‐sEVs in PNI Repair

SC‐sEVs promote peripheral nerve regeneration through multifaceted mechanisms (Figure 2), targeting both cellular and molecular components of the injury microenvironment. MSC‐sEVs can activate different signaling pathways, mediate axonal growth and remyelination, regulate neuroinflammation, and transport NTFs, thus aiding in PNI repair:

**Figure 2:**
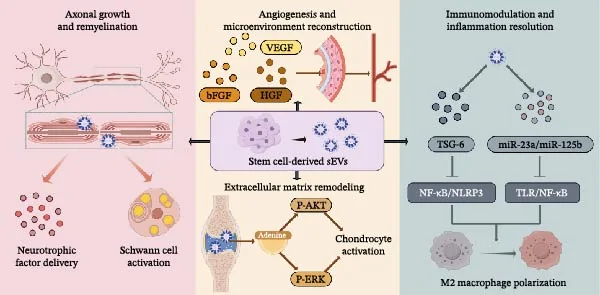
The mechanism of stem cell‐derived sEVs in PNI repair.

#### 3.2.1. Axonal Growth and Remyelination

Researchers demonstrated the presence of neural growth factors in the adipose‐derived stem cells (ADSCs) sEVs, such as BDNF, IGF‐1, NGF, FGF‐1, and GDNF [69], suggesting a possible mechanism by which sEVs bolster axon regeneration in vivo [78].

Gingival MSC‐sEVs robustly upregulate the expression of several repair SC‐related genes such as GFAP, c‐JUN, Notch1, and SOX2, inducing a proregenerative phenotype that enhances myelination and Büngner band formation [79].

#### 3.2.2. Immunomodulation and Inflammation Resolution

sEVs from lipopolysaccharide (LPS)‐preconditioned MSCs were revealed to promote macrophage polarization toward an anti‐inflammatory M2 phenotype by shuttling TNF‐stimulated gene‐6 (TSG‐6) to inactivate the NF‐κB/NLRP3 signaling axis, thus providing a potential therapeutic avenue for peripheral nerve repair [71].

sEV‑derived miRNAs (e.g., miR‐23a and miR‐125b) suppress NF‐κB signaling, possibly through the toll‐like receptor/nuclear factor kappa‐B signaling pathway, attenuating neuroinflammation and preventing secondary damage [80].

#### 3.2.3. Extracellular Matrix Remodeling

sEV‑derived CD73 induces AKT and ERK phosphorylation and activates AKT/ERK pathways in target cells such as chondrocytes, promoting chondrocyte proliferation and matrix synthesis to support nerve scaffold integrity [81].

#### 3.2.4. Angiogenesis and Microenvironment Reconstruction

Recent studies have highlighted the pro‑angiogenic potential of SC‑sEVs, which is crucial for reconstructing the vascular niche in injured nerves. SC‑sEVs are enriched with pro‑angiogenic factors such as vascular endothelial growth factor (VEGF), basic fibroblast growth factor (bFGF), and hepatocyte growth factor (HGF), as well as angiogenesis‑related miRNAs, that collectively stimulate endothelial cell proliferation and migration [82, 83]. For instance, studies have demonstrated that miR‑181b‑5p in ADSC‑sEVs promotes angiogenesis in brain microvascular endothelial cells (BMECs) by targeting and inhibiting TRPM7, thereby upregulating HIF‑1α and VEGF protein expression [84].

Despite these advances, critical gaps persist in source‐specific variability and standardization challenges. Neural crest SC‐sEVs (e.g., hfNCSCs‐sEVs) are understudied compared to MSC‐sEVs, despite their potential neural lineage specificity. The lack of consensus on isolation methods (e.g., ultracentrifugation vs. size‐exclusion chromatography) and quantification metrics hampers clinical translation.

## 4. Role and Mechanism of hfNCSCs‐sEVs in PNI Repair

hfNCSCs‐sEVs, isolated from conditioned media via ultracentrifugation [4, 85], synergistically combine the regenerative benefits of stem cells with the safety profile of sEVs. The isolation protocol, detailed in studies referenced herein, typically involves sequential centrifugation to remove cells and debris, followed by differential ultracentrifugation at high g‐force (e.g., 120,000 × g) to pellet sEVs, with final purification through washing steps [85]. This yields the preparation of nanosized vesicles that retain bioactive cargo. By circumventing risks associated with cell transplantation (e.g., immune rejection and tumorigenicity) and leveraging their nanosized structure to penetrate biological barriers, hfNCSCs‐sEVs emerge as a dual‐functional therapeutic agent. They orchestrate nerve repair through two primary mechanisms: miRNA‐mediated intercellular communication and NTF delivery, offering significant clinical potential.

### 4.1. Enhancing Perineurial Cell Proliferation and Migration

The perineurium, a protective sheath surrounding nerve fascicles, plays a pivotal role in maintaining the blood‐nerve barrier and initiating injury repair. Following PNI, perineurial cells rapidly migrate to the lesion site, forming cellular bridges that guide axonal regrowth [86].

hfNCSCs‐sEVs directly augment this process. It was shown that hfNCSCs‐sEVs promoted the proliferation of perineural cells, which exerted effects at low concentrations early on and had a more pronounced impact in later stages with higher concentrations (20 and 50 μg/mL) (*p* < 0.05) [85]. After coculture, the number of perineurial cells migrated to hfNCSCs‐sEVs wells was greater than that migrated to control wells (*p* < 0.05). With the increasing concentrations of hfNCSCs‐sEVs, the number of migrating perineurial cells was increasing at statistical significance (*p* < 0.05) [85].

These findings suggest that hfNCSCs‐sEVs accelerate perineurial bridge formation, facilitating microenvironmental support for nerve regeneration. Mechanistically, a very recent study further demonstrated that hfNCSCs‐sEVs promote perineurial cell proliferation and migration through the TGF‐β/SMAD/HAS2 signaling pathway, providing new mechanistic insights into how these vesicles orchestrate the early cellular responses following PNI [87].

### 4.2. Modulating SC Function and Neurotrophic Signaling

While perineurial cells create the initial guidance scaffold, SCs are the principal glial cells that directly support axonal regrowth and myelination. hfNCSCs‐sEVs also exert potent trophic effects on SCs. After coculture, hfNCSCs‐sEVs were internalized into SCs in vitro, subsequently accelerating the proliferation and migration of SCs and increasing NTF (e.g., BDNF and NGF) expression of SCs as well [4]. In a rat facial nerve defect model, acellular nerve allografts (ANAs) loaded with hfNCSCs‐sEVs achieved myelination thickness and motor function recovery comparable to autografts, outperforming ANA‐only controls [4].

Mechanistically, hfNCSCs‐sEVs are enriched with neural‐specific miRNAs, particularly miR‐214‐3p, which constitutes nearly 25% of the total sEV‑derived miRNA content [85]. Target prediction analyses suggest that genes regulated by these miRNAs are involved in cellular processes such as growth and migration and are associated with pathways relevant to neural regeneration, including synaptic vesicle cycling, Notch signaling, and mTOR signaling. However, it is important to emphasize that this evidence is solely correlative. The inferred mechanism is based on computational target prediction without experimental validation. Additionally, miR‐214‐3p is commonly present in sEVs derived from other stem cell types such as UCMSCs [88], raising the question of whether its enrichment in hfNCSCs‐sEVs represents a unique functional effector or simply a passive abundance bias attributable to the parental cell expression profile. Therefore, causality remains to be established, and the hypothesis that miR‐214‐3p drives nerve regeneration via Notch/mTOR pathways requires rigorous testing using loss‑of‑function (e.g., antagomir‑based inhibition or CRISPR‑mediated gene knockout) or gain‑of‑function approaches appropriately in vivo models.

Despite the promising findings described above, critical knowledge gaps remain. Whether miR‐214‐3p acts as the dominant effector or synergizes with other miRNAs (e.g., miR‐21 and miR‐23) remains unclear. hfNCSCs‐sEVs exhibit distinct miRNA profiles compared to MSC‐sEVs [89], yet their functional superiority in PNI repair lacks direct validation. Standardized protocols for hfNCSCs‐sEVs production, dosing, and delivery routes (e.g., intraneural vs. systemic) are urgently needed to advance the field.

## 5. Translational Gaps, Challenges, and Future Directions

The current in vivo evidence for hfNCSCs‑sEVs, while promising, is confined to a small‑sample rodent study of acute facial nerve injury in young rats [4]; the other available study investigated the effects on perineurial cells in vitro [85, 87]. No in vivo data exist for chronic nerve injury models. Chronic nerve injury is not simply a prolonged acute injury; it typically entails delayed repair, aged animals, or comorbidities such as ischemia, sustained inflammation, or metabolic disorders, which markedly alter the regenerative microenvironment (e.g., SC senescence, persistent neuroinflammation, and glial scar formation) and are not recapitulated in acute transection models. In contrast, MSC‑sEVs have demonstrated therapeutic efficacy in large‑animal models of non‑neural chronic tissue defects (e.g., skin, myocardium, and tendon) [90–94], yet a focused literature search reveals a striking paucity of studies evaluating SC‐sEVs in large‑animal models of critical‑length chronic peripheral nerve gaps. Thus, the translational gap for hfNCSCs‑sEVs is particularly acute: it lacks both rodent chronic nerve injury data and any large‑animal precedent.

Beyond this evidence gap, several fundamental challenges persist. The mechanistic interplay between hfNCSCs‑sEVs and endogenous repair pathways (e.g., mTOR/Notch signaling) remains poorly defined; although miR‑214‑3p is highly enriched in hfNCSCs‑sEVs and predicted to target genes in these pathways, causality has not been established by loss‑ or gain‑of‑function experiments. Standardized protocols for sEV isolation, quantification, and delivery are lacking, with no consensus on optimal methods (e.g., ultracentrifugation vs. SEC), quality control metrics, or administration routes (intraneural vs. systemic). Moreover, direct comparative studies evaluating hfNCSCs‑sEVs against other SC‑sEVs such as MSC‑sEVs are scarce, making it difficult to determine whether hfNCSCs‑sEVs offer distinct advantages for peripheral nerve repair.

To bridge these dual gaps, a stepwise research agenda is needed. First, efficacy should be established in clinically relevant rodent chronic nerve defect models, such as delayed nerve repair, aged rats, or models with comorbid metabolic disease. Second, safety and functional outcomes should be validated in large‑animal chronic nerve injury models. Third, standardized protocols for sEV isolation, characterization, and delivery must be developed, including engineering sEVs with targeting ligands or sustained‑release biomaterials such as fibrin hydrogels. Finally, phase I trials should be initiated to assess safety, optimal dosing (e.g., 10^10^ particles/cm nerve gap), and functional outcomes in human patients.

## 6. Conclusions

PNI remains a significant clinical challenge, necessitating innovative therapeutic strategies to overcome the limitations of current treatments. hfNCSCs and their sEVs have emerged as promising candidates for PNI repair, combining the advantages of pluripotency, ethical accessibility, and reduced immunogenicity. Concurrently, hfNCSCs‐sEVs offer a cell‐free alternative, leveraging their cargo of miRNAs, proteins, and signaling molecules to promote perineurial cell migration, SC activation, and extracellular matrix remodeling.

The studies highlight the efficacy of hfNCSCs‐sEVs in bridging nerve gaps and restoring motor function, rivaling autograft outcomes. However, critical challenges persist, including the need for standardized protocols for sEVs isolation and characterization, mechanistic elucidation of key pathways, and validation of safety and scalability in human trials. Future research should prioritize the refinement of mechanisms, strategies for targeted delivery, and clinical translation through phased trials. By addressing these gaps, hfNCSCs‐sEVs may offer a safe, effective, and ethically viable solution to restore neural function and improve patient outcomes.

## Author Contributions

Jiachen Zheng and Siying Fei contributed to conceptualization, data collection, and writing the draft. Yang Xu and Chi Zhang contributed to conceptualization and investigation. Haiyan Lin co‐designed, provided guidance on the study, and revised the manuscript. Fang Liu designed and principally investigated the study as well as revised the manuscript.

## Funding

This study is supported by the National Natural Science Foundation of China (NSFC) project (Grants 81571211 and 32271196) and the Naval Medical University Foundation, China (Grant JCXYXZ‐009).

## Disclosure

The funding body played no role in the design of the study and the collection, analysis, and interpretation of data and in writing the manuscript. The authors read and approved the final manuscript. All AI‐generated suggestions were critically reviewed and approved by the authors, who take full responsibility for the final content. The authors confirm compliance with the journal’s AI policy and that the AI tool used does not claim ownership or impose restrictions on the submitted content.

## Conflicts of Interest

The authors declare no conflicts of interest.

## Data Availability

All data generated or analyzed during the study appear in the article. The data that support the findings of this study are openly available in Mendeley Data at https://data.mendeley.com/datasets/9v3g5x3sw5/4.
